# Mechanisms of endocrine resistance in breast cancer: an overview of the proposed roles of noncoding RNA

**DOI:** 10.1186/s13058-015-0542-y

**Published:** 2015-03-17

**Authors:** Erin L Hayes, Joan S Lewis-Wambi

**Affiliations:** 1 Department of Cancer Biology, University of Kansas Medical Center, 3901 Rainbow Blvd, Wahl Hall East 1031, Kansas City, KS 66160 USA; 2 Department of Physiology, University of Kansas Medical Center, Kansas City, KS 66160 USA

## Abstract

Endocrine therapies such as tamoxifen and aromatase inhibitors are the standard treatment options for estrogen receptor-positive breast cancer patients. However, resistance to these agents has become a major clinical obstacle. Potential mechanisms of resistance to endocrine therapies have been identified, often involving enhanced growth factor signaling and changes in the expression or action of the estrogen receptor, but few studies have addressed the role of noncoding RNA (ncRNA). Two important types of ncRNA include microRNA (miRNA) and long noncoding RNA (lncRNA). miRNAs are small RNA molecules that regulate gene expression via translational inhibition or degradation of mRNA transcripts, while lncRNAs are larger RNA molecules that have been shown to play a role in multiple cellular maintenance functions such as protein scaffolding, chromatin looping, and regulation of mRNA stability. Both miRNA and lncRNA have recently impacted the field of breast cancer research as important pieces in the mechanistic puzzle of the genes and pathways involved in breast cancer development and progression. This review serves as an overview of the roles of miRNA and lncRNA in breast cancer progression and the development of endocrine resistance. Ideally, future experiments in the field should include identification of ncRNAs that could be potential therapeutic targets in endocrine-resistant tumors, as well as ncRNA biomarkers that facilitate more tumor-specific treatment options for endocrine-resistant breast cancer patients.

## Introduction

Breast cancer is the most commonly diagnosed cancer in the United States and is the second leading cause of cancer death. Approximately one out of every eight US women will develop invasive breast cancer over the course of her lifetime [1]. About 70% of all breast cancers express estrogen receptor (ER) alpha and belong to the molecular subtypes luminal A or luminal B [1,2]. While the exact etiology of breast cancer is not known, there is strong evidence that estrogen plays a critical role in the development and progression of the disease. ERα-positive breast cancers rely on estrogen signaling for proliferation, and hence the most effective strategy to stop or slow the growth of these hormone-sensitive tumors is to block estrogen action in the tumor using endocrine therapy. Current endocrine therapies for ERα breast cancer include: tamoxifen, the selective ER modulator that antagonizes ERα function; fulvestrant, the pure anti-estrogen that degrades/downregulates ERα; and aromatase inhibitors (AIs) (letrozole, anastrozole, and exemestane), which suppress estrogen production in peripheral tissues by blocking the aromatase enzyme. Unfortunately, the majority of patients treated with endocrine therapy eventually develop resistance, leading to disease progression and death. The mechanism by which resistance occurs is still not completely known and thus represents a major clinical problem. This review will offer information regarding the recently studied roles of noncoding RNAs (ncRNAs) in acquired endocrine resistance.

Estrogen mediates its biological effects by binding to ERα and ERβ, which are members of the nuclear receptor superfamily of ligand-inducible transcription factors [3,4]. ERα is encoded by *ESR1*, a 300 kb gene located on chromosome 6, and has six functional domains, A to F, which include both ligand-binding and DNA-binding domains, as described by Kumar and coworkers [5]. It is also important to note that there are multiple sites of phosphorylation on ERα. For instance, Ser118 phosphorylation by a mitogen-activated protein kinase (MAPK) leads to ligand-independent activation of ERα activity, and Ser167 phosphorylation by protein kinase B (Akt) can also lead to ligand-independent activation of ERα [6,7].

When estrogen binds ERα, the receptor dimerizes and translocates to the nucleus where it binds estrogen response elements in the DNA, stimulating transcription of target genes involved in cell proliferation [8]. The estrogen-activated ERα is also able to bind other transcription factors, such as Ap-1 and Sp-1, independent of estrogen response elements, where it may recruit coactivators to stimulate transcription of additional growth and survival genes [9]. Much of the molecular signaling mediated by ERα involves the MAPK and phosphoinositide 3-kinase (PI3K) pathways, the primary facilitators of cell growth and proliferation [10].

## Endocrine therapies

### Estrogen receptor antagonists

Targeting ERα using selective ER modulators and selective ER downregulators has been an effective treatment strategy for patients with ERα-positive hormone-dependent breast cancer. For the past few decades, the selective ER modulator tamoxifen has been the most widely used drug for the treatment of breast cancer, with success both as a long-term adjuvant therapy and as a preventative agent for women at increased risk for breast cancer [11,12]. Normally, estrogen-bound ERα is in a conformation that favors the recruitment of coactivators to promote transcription of target genes. However, when tamoxifen binds to the ligand-binding domain of ERα in mammary epithelium, it induces a conformation that recruits corepressors, which blocks estrogen from binding its receptor and prevents the proliferative action of ERα signaling [13]. In addition to tamoxifen, the use of selective ER downregulators, which act as pure anti-estrogens, has also been under investigation. Only one selective ER downregulator, fulvestrant, has thus far been approved for clinical use [14].

### Aromatase inhibitors

While ER antagonists have played an important role in combating ERα-positive breast cancers for the past few decades, another class of therapies has emerged – AIs. The aromatase enzyme (a cytochrome P450 heme-containing protein) is required for the synthesis of estrogen via aromatization of androgens such as testosterone [15]. Circulating levels of estrogen decrease as a woman enters menopause, since there is no longer production of estrogen by the ovaries. Thus, the local synthesis of estrogen by breast adipose tissue plays a large role in the growth and survival of ERα-positive breast tumors [16]. Inhibition of aromatase activity in these tumors is a rational treatment strategy to suppress estrogen production in peripheral tissues, thus inhibiting tumor growth.

Currently, there are three US Food and Drug Administration-approved oral AIs in clinical use for the treatment of postmenopausal women with hormone receptor-positive breast cancer. These AIs can be divided into two categories: steroidal AIs (exemestane) and nonsteroidal AIs (anastrozole, letrozole). There has been great interest in the development of AIs since it has been observed in clinical trials that they can be more tolerable than tamoxifen, and are usually more effective or equivalently effective in clinical response rate and median time to progression [17]. Indeed, the third-generation AIs have all been shown to suppress circulating estrogen levels in postmenopausal breast cancer patients by more than 97% [18,19].

## Resistance to endocrine therapies

### Mechanisms of tamoxifen resistance

**Figure 1:**
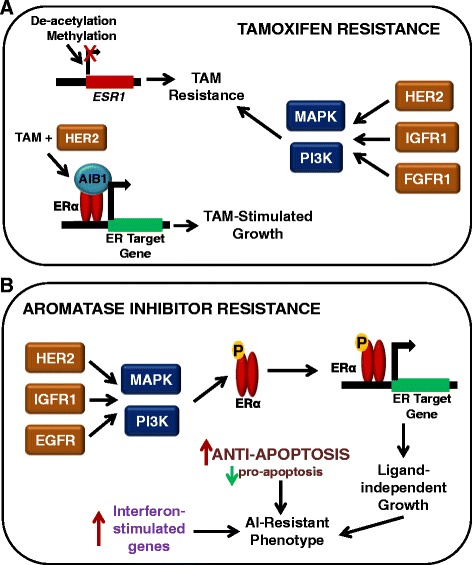
**Mechanisms of endocrine resistance in breast cancer cells. (A)** Mechanisms of tamoxifen (TAM) resistance may involve the loss of estrogen receptor (ER) alpha expression, which can be achieved by methylation of CpG islands or histone deacetylase activity in the *ESR1* promoter. Tamoxifen-resistant growth is also stimulated by the upregulation of growth factor signaling pathways (HER2, IGFR1, and FGFR1) and subsequent activation of the mitogen-activated protein kinase (MAPK) cascade or phosphoinositide 3-kinase (PI3K) pathway. Finally, tamoxifen has even been shown to stimulate the growth of breast cancer cells when bound to certain coactivators, such as AIB1, and this is especially true in HER2-expressing cells. **(B)** The mechanisms of aromatase inhibitor (AI) resistance share similarities with tamoxifen resistance, especially in terms of growth factor pathway upregulation. The enhanced activity of growth factors such as MAPK can result in estrogen-independent phosphorylation and activation of ERα. In addition to growth factor signaling, interferon response genes and anti-apoptotic proteins have also been shown to have increased expression in AI-resistant cells. AIB1, amplified in breast cancer 1; FGFR1, fibroblast growth factor receptor 1; HER2, human epidermal growth factor receptor 2; IGFR1, insulin-like growth factor receptor 1.

Many of the key pathways involved in tamoxifen resistance involve growth factors, such as human epidermal growth factor receptor 2 (HER2) (Figure 1A). The growth of the tamoxifen-resistant cell model MCF-7/HER2-18 (HER2 overexpressing) is increased with treatment of tamoxifen, revealing a crosstalk between HER2 and ERα [20]. Likewise, early *in vivo* studies of breast tumors by Gottardis and Jordan revealed that, in the process of acquiring tamoxifen resistance, tumors may gain the ability to grow in a tamoxifen-stimulated manner [21]. There is strong evidence that the ability of tamoxifen to function as an agonist or an antagonist is dependent on whether it recruits coactivators or corepressors to the ERα transcription complex [22]. Perhaps the most studied coregulator of ERα is the amplified in breast cancer 1 (AIB1) protein. Increased expression of AIB1 correlates with tamoxifen resistance since AIB1 expression contributes to the agonistic activity of tamoxifen – especially in the presence of HER2 (Figure 1A) [23].

In addition to HER2 signaling, the growth factor receptors insulin-like growth factor receptor 1 and fibroblast growth factor receptor 1 can activate the MAPK and PI3K pathways, which have been shown to confer tamoxifen unresponsiveness [24,25]. Altered expression of ERα also contributes to the development of tamoxifen resistance. Since ERα is the target of tamoxifen treatment, lack of ERα expression is known to result in resistance. Hypermethylation of CpG islands and histone deacetylase activity in the *ESR1* promoter (Figure 1A) are similar to the absence of ERα because these can inactivate the gene so the cells express much less ERα [26].

Resistance to tamoxifen can also arise from dysregulated metabolism of the drug. In the liver, cytochrome P450 enzymes CYP2D6 and CYP3A4 convert tamoxifen to its active metabolites 4-hydroxytamoxifen and endoxifen, which both have 30-fold to 100-fold higher potency to inhibit estrogen-dependent proliferation than tamoxifen [27]. Polymorphisms in the cytochrome P450 proteins, especially CYP2D6, have been associated with poor metabolic activity, and are associated with worse clinical outcome after tamoxifen treatment [28,29]. In addition, it is also possible that altered cellular accumulation of tamoxifen and its metabolites – potentially through the induction of efflux transporters such as P-glycoprotein/multi-drug resistance protein 1 (MDR1) – might influence a patient’s response to tamoxifen [30]. Notably, P-glycoprotein expression has been associated with a shorter overall survival for tamoxifen-treated patients, but its use as a prognostic marker is still under investigation [31,32].

### Mechanisms of aromatase inhibitor resistance

There are several pathways implicated in the acquired AI-resistant phenotype. These include the MAPK [33], epidermal growth factor receptor [34], and PI3K pathways (Figure 1B) [35]. ERα has also been shown to play a role in AI resistance, in the form of a constitutively active ligand-independent mutant ERα [36], via different genome binding patterns [37], or simply by modified expression levels [38]. In fact, one study reveals that ligand-independent ERα activation is required for the development of an AI-resistant phenotype in the aromatase-overexpressing MCF-7aro cell line [39]. The phosphorylation of ERα by MAPK (Ser118) and Akt (Ser167) is often essential for the ligand-independent action of ERα, as discussed previously (Figure 1B) [6,7].

Since the mechanism by which AIs induce death of ER-positive breast cancer cells often involves apoptosis, a disturbance in the balance of pro-apoptotic and anti-apoptotic genes could also play a role in resistance to AI treatment (Figure 1B) [40]. Indeed, this imbalance has been shown in an aromatase-expressing MCF-7 cell line with a mutant ERα gene (K303R) [41]. The K303R mutation was shown to cause resistance to both tamoxifen and the AI anastrozole, and these K303R MCF-7/Aro cells have an increase in the Bcl-2 (anti-apoptotic)/Bax (pro-apoptotic) ratio, which is further exacerbated upon treatment with anastrozole [41].

The clonally selected long-term estrogen-deprived (LTED) models MCF-7:5C [42,43] and MCF-7:2A [44,45], used in our laboratory, have revealed some of the significant genetic reprogramming that occurs when the breast tumor cells are deprived of estrogen in the long term (>1 year) [46]. Notably, recent findings from our laboratory have identified a critical role for interferon-stimulated genes, in particular interferon-induced transmembrane protein 1 (*IFITM1*), which has been shown to be markedly overexpressed (>25-fold) in AI-resistant MCF-7:5C breast cancer cells and AI-resistant breast tumors. Interestingly, we have found that overexpression of IFITM1 is strongly associated with enhanced cell survival, proliferation, and invasiveness of AI-resistant cells and that knockdown of IFITM1 induces cell death and blocks the ability of the resistant cells to migrate and invade [47].

## Noncoding RNAs and breast cancer

The development and progression of breast cancer are affected by many factors, most of which involve a change in expression of certain genes. Of the mechanisms for regulating gene expression, ncRNAs have proven to be integral to the cell in recent studies [48]. ncRNAs include microRNA (miRNA), long noncoding RNA (lncRNA), transfer RNA, ribosomal RNA, and small nucleolar RNA. Two forms of ncRNA that are especially important for regulation of gene expression in cells are miRNA and lncRNA.

### microRNAs and breast cancer

**Figure 2:**
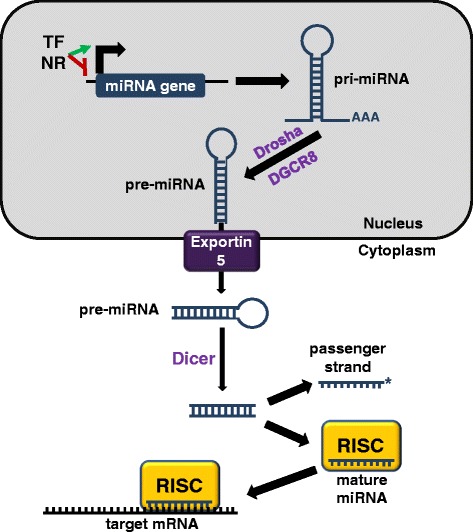
**Standard pathway by which microRNAs are processed and loaded onto RISC to regulate gene expression. **Regulation of microRNA (miRNA) expression is controlled at the miRNA promoter by transcription factors (TF) and nuclear receptors (NR). After transcription, the pri-miRNA is processed inside the nucleus by Drosha and DGCR8 to form pre-miRNA – a hairpin miRNA. Exportin 5 exports the pre-miRNA from the nucleus into the cytoplasm where it gets cleaved further by Dicer, resulting in a short double-strand piece of RNA. These strands are separated into the passenger strand, which often gets degraded, and the mature strand, which is loaded onto RNA-induced silencing complex (RISC) for action on target mRNA. If the seed sequence (base pairs 2 to 7) of the mature miRNA is complementary to the mRNA, the transcript is degraded. However, if there is not perfect complementarity between the miRNA seed sequence and its target mRNA, the result is inhibition of translation. DGCR8, DiGeorge syndrome chromosomal region 8.

miRNAs are small RNA molecules of 18 to 22 base pairs that regulate the expression of target mRNAs by inhibiting translation or degrading the transcripts [49]. After miRNA genes are initially transcribed by RNA polymerase II, a process regulated by transcription factors and nuclear receptors, the transcripts undergo significant processing both in the nucleus and cytoplasm (Figure 2) [50]. First, the pri-miRNA sequence is cleaved in the nucleus by Drosha, a class 2 RNase III enzyme that works in conjunction with DiGeorge syndrome chromosomal region 8 (DGCR8). The resulting pre-miRNA hairpin is exported from the nucleus to the cytoplasm via Exportin 5 where it is further cleaved by Dicer, another member of the RNase III family, resulting in a short double-strand piece of RNA. The two strands of the RNA then separate into the passenger strand, which often gets degraded, and the mature miRNA strand, which binds to argonaute (Ago) proteins to form the RNA-induced silencing complex [49]. The miRNA of the RNA-induced silencing complex guides the complex to a complementary mRNA 3′ untranslated region. Complementarity of the miRNA seed sequence (base pairs 2 to 7) to the target mRNA stimulates Ago2 in the RNA-induced silencing complex to degrade the mRNA [51]. However, if there is only partial complementarity, the translation of the mRNA will be inhibited.

Expression profiles of miRNA in breast tumor samples have been correlated with biopathologic features such as hormone receptor status and proliferation index, and are used to distinguish between basal and luminal subtypes [52,53]. For example, miRNAs overexpressed in basal, ERα-negative primary breast cancers include miR-150, which has been shown to promote breast cancer growth [54], and miR-135b, which correlates with early metastasis of breast cancer cells [55]. miRNAs overexpressed in luminal, ERα-positive breast cancers include miR-126 and miR-10a, which are associated with an increase in patients’ relapse-free time after tamoxifen treatment [56].

Beyond the use of expression profiles, specific miRNAs have been associated with regulation of genes involved in breast cancer. The let-7 miRNA family has been shown to regulate the self-renewal capacity of breast tumor-initiating cells derived from cell lines and primary patient tumors by inhibition of *HRAS* and high mobility group AT-hook2 (*HMGA2*) – genes involved in self-renewal and differentiation, respectively [57]. miR-21, which targets phosphatase and tensin homolog (*PTEN*), was identified to be upregulated in primary patient samples of invasive breast cancer compared with normal breast tissue by miRNA *in situ* hybridization staining [58]. miR-373 and miR-520c are considered metastasis-promoting miRNAs and are shown to be upregulated in lymph node metastases compared with primary tumor samples [59]. Promotion of tumor invasion and metastasis by miR-373 and miR-520c is probably achieved via suppression of the *CD44* gene, which codes for a hyaluronan receptor and has been identified as a metastasis suppressor in breast cancer [60].

For a more comprehensive look at the miRNAs involved in breast cancer, the reader should refer to reviews by O’Day and Lal and by Singh and Mo [61,62].

### Long noncoding RNAs and breast cancer

**Figure 3:**
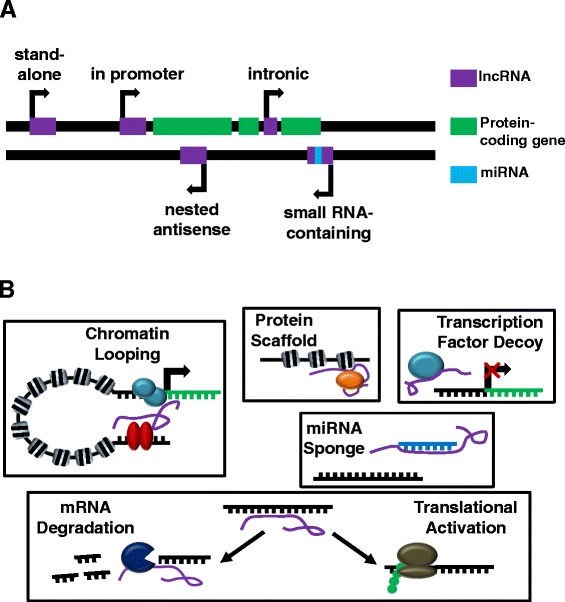
**Location of long noncoding RNAs in the genome and roles of long noncoding RNAs in regulation of cellular processes. (A)** Long noncoding RNA (lncRNA) genes reside in various genomic locations, such as in the promoters, enhancers, introns, or anti-sense coding regions of genes, and can also be in their own stand-alone position in the genome. These lncRNA genes sometimes contain small RNA genes, like microRNA (miRNA), that are spliced out of the lncRNA after transcription. **(B)** The actions of lncRNAs affect many cellular processes. lncRNAs may serve as scaffolds to bring nuclear receptors in contact with promoters of their target genes via chromatin looping, or they may recruit an epigenetic modifier to the chromatin. They can also bind proteins, such as transcription factors, to prevent their binding to DNA – similar to their mechanism of miRNA inhibition. Among the effects lncRNAs have on mRNA, translational activation and maintenance of mRNA stability are also important.

lncRNAs are ncRNA transcripts longer than 200 base pairs that are transcribed from various genomic locations, such as in the promoters, enhancers, introns, or anti-sense coding regions of genes, or in their own stand-alone position in the genome (Figure 3A) [63]. Unlike miRNAs whose primary role is to repress the translation of their mRNA targets, lncRNAs have been shown to act as protein–DNA or protein–protein scaffolds, miRNA sponges, and protein decoys, as well as regulators of translation (Figure 3B) [64]. While previously thought of as junk DNA, lncRNAs are now regarded as an important part of the cell’s gene regulation machinery, controlling cell cycle, apoptosis, and differentiation [65-67].

Some investigators have begun to identify lncRNAs whose expression is associated with aberrant signaling or unregulated survival of cancer cells. According to the lncRNADisease Database [68], there are 16 lncRNAs known to play a role in breast cancer including H19, growth arrest-specific 5 (GAS5), homeobox antisense intergenic RNA (HOTAIR), and breast cancer anti-estrogen resistance 4 (BCAR4) [69-72].

The lncRNA H19, present on the maternal allele, normally plays a role in imprinted regions of the genome to silence insulin-like growth factor 2 (*IGF2*) [73]. H19 was said to have an oncogenic role in breast cancer cells in 2002, but has since been found to exhibit tumor suppressive action *in vivo* [69,74]. Hormonal regulation of H19 is important, as estradiol is able to stimulate H19 transcription in MCF-7 breast cancer cells, while tamoxifen inhibits this transcription [75].

GAS5 expression is downregulated in breast cancer samples relative to adjacent unaffected normal breast tissue, and has a distinct tumor suppressive role in breast cancer by inducing apoptosis and suppressing cell proliferation [70]. GAS5 acts primarily by preventing the glucocorticoid receptor from binding target DNA. Despite additional interactions with the androgen receptor and progesterone receptor, results show that GAS5 does not bind the ER [76].

HOTAIR was first identified as an lncRNA that regulates the homeobox D (*HOXD*) cluster by tethering the polycomb repressor complex 2 (PRC2) protein to the DNA at this site. PRC2 is able to promote histone H3K27 trimethylation and subsequent repression of transcription at the *HOXD* cluster, thereby preventing differentiation and leading to an invasive cellular phenotype [77]. Overexpression of HOTAIR has been associated with enhanced metastasis and invasion of breast cancer cells and can be used as a predictor of overall survival and progression-free survival [71]. A potential explanation for HOTAIR overexpression in breast cancer could be related to the presence of estrogen response elements in the HOTAIR promoter, leading to estradiol-induced HOTAIR expression [78].

Of the known lncRNAs associated with breast cancer, BCAR4 is most noteworthy for its role in endocrine resistance (see Long noncoding RNAs and tamoxifen resistance).

## Noncoding RNAs and endocrine resistance

### microRNAs and tamoxifen resistance

**Figure 4:**
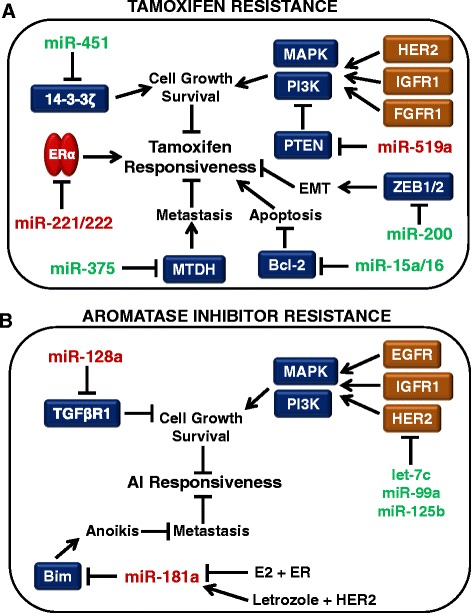
**Role of microRNA in endocrine resistance.** microRNAs (miRNAs) that regulate the growth, survival, apoptosis, epithelial-to-mesenchymal transition (EMT), and metastasis of breast cancer cells are implicated in the loss of responsiveness to endocrine therapies. miRNAs that are upregulated in endocrine resistance (red) could potentially be targets of RNA interference therapies, while miRNAs that are downregulated in endocrine resistance (green) could be targets of a replacement therapy in endocrine-resistant breast tumors. **(A)** miRNAs involved in tamoxifen resistance. **(B)** miRNAs involved in aromatase inhibitor (AI) resistance. Bim, Bcl-2-like 11; EGFR, epidermal growth factor receptor; ER, estrogen receptor; E2, 17β-estradiol; FGFR1, fibroblast growth factor receptor 1; HER2, human epidermal growth factor receptor 2; IGFR1, insulin-like growth factor receptor 1; MAPK, mitogen-activated protein kinase; MTDH, metadherin; PI3K, phosphoinositide 3-kinase; PTEN, phosphatase and tensin homolog; TGFR1β, transforming growth factor beta receptor 1; ZEB1/2, zinc finger E box-binding homeobox 1/2.

The miRNAs associated with endocrine resistance have been explored, and this effort has primarily focused on the differential expression of miRNAs in tamoxifen-resistant cells. miRNAs that inhibit ERα, such as miR-221/222, are implicated in resistance to anti-ERα therapies. One study showed that ectopic expression of miR-221 or miR-222 was enough to decrease ERα protein expression in MCF-7 and T47D breast cancer cells, and this led to the cells acquiring resistance to tamoxifen (Figure 4A) [79]. In addition, silencing of miR-221 and miR-222 in ERα-negative, endocrine-resistant MDA-MB-468 breast cancer cells has been shown to increase ERα expression and sensitize cells to tamoxifen-induced apoptosis [79].

In addition to targeting ERα, miRNAs associated with tamoxifen resistance have been shown to regulate genes related to cell survival and metastasis. Following *in vitro* treatment of MCF-7 cells with tamoxifen there is an increase in cell survival factor 14-3-3ζ [80]. miR-451, which normally targets the anti-apoptotic factors PI3K/Akt and 14-3-3ζ, has very low expression in tamoxifen-resistant MCF-7 cells, which could contribute to the survival of these cells after tamoxifen treatment (Figure 4A). In fact, re-expression of miR-451 has been shown to decrease cell proliferation and colony formation, as well as reduce HER2, epidermal growth factor receptor, and MAPK levels, followed by a restored sensitivity to tamoxifen in resistant MCF-7 cells [80]. There is also evidence that miR-451 expression is reduced in doxorubicin-resistant MCF-7 breast cancer cells and that it regulates the drug transporter P-glycoprotein/MDR1 in these cells [81]. Both tamoxifen and 4-hydroxytamoxifen are known to bind to P-glycoprotein, and endoxifen has been shown to be a substrate of this transporter, thus suggesting the importance of this efflux transporter to tamoxifen therapy [82,83].

In another tamoxifen-resistant cell model (MCF-7/TAMR), miR-375 was identified as one of the top downregulated miRNAs. Re-expression of miR-375 was enough to reverse the tamoxifen-resistant phenotype via repression of metadherin (*MTDH*), a metastasis-promoting cell surface protein (Figure 4A) [84]. Expression of MTDH and miR-375 were inversely correlated in primary breast cancer samples, and survival data from tamoxifen-treated patients revealed that higher expression of MTDH was associated with a shorter disease-free survival and higher risk of relapse [84]. Another recent study of miRNA in the tamoxifen-resistant MCF-7 TamR cells has revealed upregulation of the C19MC cluster, a primate-specific cluster of 19 miRNAs, with miR-519a being most highly overexpressed [85]. miR-519a was shown to directly target the tumor suppressor genes *CDKN1A* (P21), *PTEN*, and *RB1*, allowing for the enhanced signaling of the PI3K growth and survival pathway in tamoxifen-resistant cells (Figure 4A).

MCF-7 cells expressing an oncogenic isoform of HER2 (MCF-7/HER2Δ16) evade tamoxifen treatment by upregulating the expression of the anti-apoptotic factor Bcl-2 and downregulating the expression of miR-15a/16, which inhibit expression of Bcl-2 [86]. Treatment of the MCF-7/HER2Δ16 cells with exogenous miR-15a/16 decreased tamoxifen-induced Bcl-2 levels and re-sensitized the cells to tamoxifen-induced cell death [86]. These MCF-7/HER2Δ16 cells also present decreased expression of miR-342, which was shown to regulate expression of genes involved in breast tumor cell cycle progression, such as cyclin B1, p53, and breast cancer 1 (BRCA1) [87]. miR-342 expression was also shown to be downregulated in tamoxifen refractory human breast tumors, which confirms its clinical relevance [87].

Other miRNAs that play a role in tamoxifen resistance include members of the miR-200 family – miR-200a, miR-200b, and miR-200c, all of which inhibit the expression of zinc finger Ebox-binding homeobox 1/2 (*ZEB1/2*) [88]. The miR-200 family has been shown to be expressed at low levels in tamoxifen-resistant LY2 breast cancer cells compared with tamoxifen-sensitive MCF-7 parental cells (Figure 4A). ZEB1/2-mediated transcriptional repression of E-cadherin is necessary for epithelial-to-mesenchymal transition, so the upregulation of ZEB1/2 could contribute to the invasiveness of tamoxifen-resistant breast cancer cells. Overexpression of the miR-200 family members has been shown to inhibit cell migration and reverse epithelial-to-mesenchymal transition [88].

In a study of 235 ERα-positive breast cancer specimens, high expression of miR-26a was significantly associated with clinical benefit and prolonged time to progression. A combination of high miR-26a expression and low expression of the miR-26a targets cyclin-dependent kinase 1 (*CDC2*) and cyclin E1 (*CCNE1*) was associated with favorable outcome after tamoxifen treatment [89]. Another study measured the expression of five miRNAs in 246 ERα-positive primary breast cancer tumors from patients treated with tamoxifen for advanced disease and found that high expression of miR-30a, miR-30c, and miR-182 was significantly associated with tamoxifen sensitivity and longer progression-free survival [90]. miR-30c was the most significant and independent predictor of clinical benefit, and correlated positively with ERα and inversely with epidermal growth factor receptor [90]. Lastly, analysis of miRNA expression in matched samples from ERα-positive breast cancer patients treated with tamoxifen indicated that high expression of miR-126 and miR-10a were associated with an increase in patients’ relapse-free time after tamoxifen treatment [56]. Additionally, miR-126 has been shown to suppress metastasis of breast cancers in subtype-specific mechanisms that involve inhibiting infiltration of mesenchymal stem cells and inflammatory monocytes [91].

### Long noncoding RNAs and tamoxifen resistance

The lncRNA BCAR4 was discovered in a functional screen of ZR-75-1 breast cancer cells to identify mechanisms of anti-estrogen resistance [92]. Overexpression of BCAR4 in tamoxifen-sensitive ZR-75-1 cells blocked the anti-proliferative effects of tamoxifen, and BCAR4 has since been shown to be a clinically relevant biomarker for increased invasiveness and tamoxifen resistance [72,92]. The role of BCAR4 in tamoxifen resistance relies on the coexpression of HER2, but is independent of ERα [93,94]. A HER2 inhibitor may thus be ideal for those patients whose tumors are resistant to traditional endocrine therapy due to high levels of BCAR4, and are accompanied by HER2 overexpression. However, if a method of RNA interference is developed to diminish levels of BCAR4 in breast cancer patients, this could also be a rational therapy. When it is not present at high levels in breast tumors, BCAR4 is normally found only in the human placenta and oocyte, diminishing the side effects of such a therapy [93].

### microRNAs and aromatase inhibitor resistance

The topic of miRNAs involved in AI resistance is newer than that of miRNAs in tamoxifen resistance, but recent studies have started to shed light on the field. Masri and colleagues conducted a miRNA expression analysis for four AI-resistant cell models, all derived from aromatase-overexpressing MCF-7aro cells [95]. MCF-7aro cells were treated with testosterone and each of three AIs until they acquired resistance. An LTED cell line (LTEDaro) was also derived from the MCF-7aro cells for comparison. When compared with LTEDaro cells and cells treated only with testosterone, letrozole-resistant cells overexpressed miR-128a – suggesting miR-128a plays a role in AI resistance. miR-128a is associated with breast cancer aggressiveness, and is involved in regulation of transforming growth factor beta receptor 1 (Figure 4B) [96]. Inhibition of miR-128a in the letrozole-resistant cells led to resensitization to transforming growth factor beta growth-inhibitory effects [95]. It is also interesting to note that the expression profiles for the cells resistant to the steroidal AI (exemestane) and the nonsteroidal AIs (letrozole, anastrozole) were distinct from one another, and the AI-resistant cells were distinct from the LTEDaro model of AI resistance, which supports the idea that multiple mechanisms of acquired resistance to AIs may exist [95].

Unpublished data from our laboratory have given additional insight into the miRNAs that may be involved in the AI-resistant phenotype. Next-generation sequencing of miRNA in MCF-7 cells, MCF-7:5C (LTED) cells, and MCF-7:2A (LTED) cells has revealed over 120 miRNAs whose expression levels are altered in both the MCF-7:5C and MCF-7:2A cells compared with the parental MCF-7 cells. One of the most interesting results is that both MCF-7:5C and MCF-7:2A cells show an increase in miR-181a, which targets Bcl-2-like 11 (Bim), a pro-apoptotic protein involved in anoikis (cell death) after breast cancer cells detach from the basement membrane in preparation for metastasis (Figure 4B) [97]. Recent work from Angela Brodie’s laboratory indicates a HER2-dependent upregulation of miR-181a in letrozole-resistant ERα-negative/HER2-positive breast cancer cells, and indicates that miR-181a expression is increased in ERα-negative/HER2-positive and ERα-positive/HER2-positive clinical samples [98]. Several miRNAs have been described as estrogen responsive, and this information is important when studying estrogen-deprived cells in these AI-resistant tumors [99]. Thus, it is worth noting that miR-181a is downregulated upon estrogen treatment, which might explain its overexpression in the LTED cells [100].

In addition to the confirmed role of miR-128a, Masri and colleagues also briefly mentioned the downregulation of miR-125b in the letrozole-resistant, anastrozole-resistant, and exemestane-resistant cells compared with the LTEDaro cells [95]. A recent study in MCF-7:2A (LTED) cells confirms downregulation of miR-125b in this AI-resistant LTED model and also indicates relevance of the other members of its miRNA cluster [101]. The let-7c/miR-99a/miR-125b miRNA cluster encoded within the *LINC00487* gene was shown to be downregulated in MCF-7:2A cells compared with MCF-7 parental cells. A luciferase reporter assay confirmed that let-7c and miR-125b bind the HER2 3′ untranslated region, but it was revealed that while miR-125b directly regulates HER2 expression via the binding of HER2 3′ untranslated region, let-7c regulation of HER2 is indirect via the inhibition of Dicer, one of the proteins involved in miRNA function [101]. Upon analysis of clinical data from The Cancer Genome Atlas, it was clear that let-7c and HER2 were inversely correlated in luminal A breast tumors and that low expression of the let-7c/miR-99a/miR-125b cluster was associated with worse overall survival compared with patients who had high expression of this cluster [101].

Proposed roles of miRNA in AI resistance can be also determined by focusing on miRNAs that have shown importance in estrogen-independent growth and survival or are differentially expressed following anti-estrogen therapy. An *in vivo* selection process using a miRNA library revealed six miRNAs that were shown to confer estrogen-independent growth and increased phosphorylated-Akt in MCF-7 breast cancer cells [102]. Of these six, miR-101 was capable of promoting estrogen-independent growth, while the other five were not. Further characterization of miR-101 revealed that it inhibits expression of membrane-associated guanylate kinase (MAGI-2), a protein necessary for PTEN activity [102]. Expression of miR-101 is thus important for estrogen-independent growth mediated by the PI3K/Akt pathway, which could be important for resistance to both tamoxifen and AIs.

In a study to identify estrogen-regulated miRNAs, a group of miRNAs was shown to have elevated expression upon combined treatment of tamoxifen and exemestane, including miR-21, miR-181b, miR-26a/b, miR-27b, and miR-23b [100]. As discussed earlier, miR-21 is upregulated in primary patient samples of invasive breast cancer compared with normal breast tissue, and miR-26a is associated with favorable outcome after tamoxifen treatment [58,89]. While these miRNAs have not been directly implicated in AI resistance, their overexpression after treatment makes them candidates for a role in survival of AI-resistant cells. Likewise, a study of miRNA expression in MCF-7 cells revealed several miRNAs that are increased following letrozole treatment [103]. Perhaps the most interesting result was the upregulation of let-7f upon treatment with letrozole, as it inhibits the expression of the aromatase gene (*CYP19A1*) [103]. This study may reveal a mechanism of resistance similar to that of tamoxifen resistance – loss of the inhibitor’s target. Just as tamoxifen resistance occurs in conjunction with absence of ERα, resistance to AIs may eventually develop due to pronounced loss of aromatase expression due to upregulation of certain miRNAs upon treatment.

### Long noncoding RNAs and aromatase inhibitor resistance

There have been few studies that have focused specifically on the role of lncRNA in AI resistance. Nevertheless, there is some evidence that the lncRNAs regulating steroid receptors such as ERα may play a role in resistance. It has been shown that the lncRNA steroid receptor RNA activator 1 (SRA1) acts as a coactivator of ERα, and this action depends on the phosphorylation of ERα at Ser118 [104]. When this phosphorylation event occurs via the kinase action of MAPK (a protein that is often upregulated in AI-resistant cells), it is associated with estrogen-independent activation of ERα [6]. This putative mechanism of resistance is similar to that of AIB1 and tamoxifen resistance – just as AIB1 coactivates ERα in the presence of tamoxifen and this is enhanced by the presence of HER2, SRA1 could potentially coactivate ERα in an AI-induced estrogen-deprived environment, and this requires MAPK activity.

The scaffolding lncRNAs prostate cancer associated noncoding RNA 1 (PRNCR1) and prostate-specific transcript 1 (PCGEM1) have previously been reported to play a role in transcription of androgen receptor target genes in the androgen-resistant prostate cancer cell lines CWR22Rv1 and LNCaP-cds1/2 via a chromatin-looping mechanism involving the androgen receptor [105]. A more recent study by Prensner and colleagues, however, refutes that PCGEM1 and PRNCR1 interact with the androgen receptor and that either gene is a component of androgen receptor signaling or is involved in castration-resistant prostate cancer [106]. There were differences between the two studies, which might partially explain the conflicting observations reported. Prensner and coworkers analyzed castration-resistant prostate tumor samples that were collected from high-risk prostate cancer patients but they did not evaluate castration-resistant prostate cancer cell lines, whereas the study by Yang and coworkers evaluated castration-resistant prostate tumor samples as well as castration-resistant prostate cancer cell lines. Overall, despite the inconclusive results from these two studies, they still offer insight into a possible chromatin-looping mechanism by which lncRNAs could facilitate the transcription of ERα target genes in the absence of estrogen in breast cancer cells that have acquired AI resistance.

## Conclusions

In summary, there are numerous ncRNAs shown to be involved in acquired resistance to endocrine therapies. ncRNAs provide an exciting avenue of gene regulation that has not yet been fully explored. As we uncover the miRNAs and lncRNAs involved in specific disease states, such as resistance to breast cancer treatments, it will be possible to use these RNAs as both therapeutic targets and biomarkers.

Regarding therapeutic approaches, oncogenic ncRNAs that contribute to the progression of disease would need to be eliminated via RNA interference, while tumor-suppressive ncRNAs may be part of replacement therapies. There are over 20 RNA interference-based therapies employing various methods of small interfering RNA delivery currently in phase I clinical trials for the treatment of diseases such as viral infections, hereditary disorders, and cancer [107]. The only RNA replacement clinical trial to date began in April 2013 as a strategy to deliver miR-34, a tumor-suppressive miRNA that regulates expression of *BCL-2* and *MYC*, to patients with liver-based cancers [108]. Although there are not yet published results on this MRX34 treatment, a recent update revealed that MRX34 has a manageable safety profile with only one incidence of a dose-limiting toxicity [109].

With the use of innovative technologies, it is now possible to use ncRNAs as biomarkers and compile biomarker panels for diagnosis and prognosis of diseases, including cancer. Circulating miRNAs are ideal for clinical use, since they are highly stable and can be detected by a non-invasive manner in a blood sample. Circulating miRNA levels in breast cancer patients have been studied at diagnosis, in early stage tumors, after surgical resection, following chemotherapy/radiation treatments, and following metastatic relapse – all to understand the unique miRNA profiles throughout the progression of breast cancer [110-112]. Because of the low abundance of miRNAs in the blood, the use of powerful detection methods such as high-throughput sequencing will need to be employed in clinical settings. A more complete picture of the differentially expressed regulatory RNAs is crucial for the development of these therapeutic strategies and biomarker signatures, especially in a disease as complex as breast cancer.

## Abbreviations

Ago: Argonaute
AI: Aromatase inhibitor
AIB1: Amplified in breast cancer 1
Akt: Protein kinase B
BCAR4: Breast cancer anti-estrogen resistance 4
ER: Estrogen receptor
GAS5: Growth arrest-specific 5
HER2: Human epidermal growth factor receptor 2
HOTAIR: Homeobox antisense intergenic RNA
HOXD: Homeobox D
IFITM1: Interferon-induced transmembrane protein 1
lncRNA: Long noncoding RNA
LTED: Long-term estrogen-deprived
MAPK: Mitogen-activated protein kinase
MDR1: Multi-drug resistance protein 1
miRNA: microRNA
MTDH: Metadherin
ncRNA: Noncoding RNA
PCGEM1: Prostate-specific transcript 1
PI3K: Phosphoinositide 3-kinase
PRC2: Polycomb repressor complex 2
PRNCR1: Prostate cancer associated noncoding RNA 1
PTEN: Phosphatase and tensin homolog
SRA1: Steroid receptor RNA activator 1
ZEB1/2: Zinc finger E box-binding homeobox 1/2
